# Coulomb nanoradiator-mediated, site-specific thrombolytic proton treatment with a traversing pristine Bragg peak

**DOI:** 10.1038/srep37848

**Published:** 2016-11-29

**Authors:** Jae-Kun Jeon, Sung-Mi Han, Soon-Ki Min, Seung-Jun Seo, Kyuwook Ihm, Won-Seok Chang, Jong-Ki Kim

**Affiliations:** 1 Departments of Biomedical Engineering, Catholic University of Daegu, School of Medicine, Daegu, Korea; 2 Anatomy, and Diagnostic Imaging, Catholic University of Daegu, School of Medicine, Daegu, Korea; 3 Catholic University of Daegu, School of Medicine, Daegu, Korea; 4 Pohang Accelerator Laboratory, Pohang, Korea

## Abstract

Traversing proton beam-irradiated, mid/high-Z nanoparticles produce site-specific enhancement of X-ray photon-electron emission via the Coulomb nanoradiator (CNR) effect, resulting in a nano- to micro-scale therapeutic effect at the nanoparticle-uptake target site. Here, we demonstrate the uptake of iron oxide nanoparticles (IONs) and nanoradiator-mediated, site-specific thrombolysis without damaging the vascular endothelium in an arterial thrombosis mouse model. The enhancement of low-energy electron (LEE) emission and reactive oxygen species (ROS) production from traversing proton beam-irradiated IONs was examined. Flow recovery was only observed in CNR-treated mice, and greater than 50% removal of the thrombus was achieved. A 2.5-fold greater reduction in the thrombus-enabled flow recovery was observed in the CNR group compared with that observed in the untreated ION-only and proton-only control groups (*p* < 0.01). Enhancement of the X-ray photon-electron emission was evident from both the pronounced Shirley background in the electron yield and the 1.2- to 2.5-fold enhanced production of ROS by the proton-irradiated IONs, which suggests chemical degradation of the thrombus without potent emboli.

Thrombotic occlusion is the major pathological event in blood-clotting disorders such as myocardial infarction, ischemic stroke, pulmonary embolism, thrombosed haemodialysis access sites, and potentially, atherosclerosis^1^. Tissue plasminogen activator (tPA)-based thrombolytic therapy is not always safely efficacious in these diseases because of fibrinolysis-mediated re-occlusion, endogenous tolerance to tPA, and major bleeding complications^2^. Vascular inflammation, disruption of the blood-brain barrier, and secondary thrombosis limit the therapeutic window for the treatment of stroke thrombolysis with tPA to 3 hours^3^. Platelet-rich emboli occlusions in replanted tissues and free flaps often lead to failure in microvascular surgery^4^. Distal embolization from the disrupted plaque^5^,^6^ is one difficulty associated with angioplasty and other focal treatment techniques. Therefore, novel non-invasive thrombolytic treatments without haemorrhagic and embolic complications are desirable, and the Coulomb nanoradiator (CNR) effect is potentially applicable to induce site-specific thrombolysis in these cases.

Iron oxide nanoparticles (IONs) are promising indicators of thrombus-associated inflammation^7^,^8^,^9^. They can be taken up in thrombi via macrophages or entrapped in the thrombus mesh^10^,^11^, enabling ION-based magnetic resonance imaging (MRI) and thrombosis imaging^12^.

The CNR effect is defined as the production of burst emissions of fluorescent X-rays and low-energy electrons (LEEs) via Auger cascades and interatomic/intermolecular Coulomb decay (ICD) paths^13^,^14^ from mid/high-Z nanoparticles under irradiation by a high-energy ion beam^15^,^16^,^17^,^18^.

In this work, instead of the conventional spread-out Bragg-peak (SOBP), which is not sufficiently precise to deliver conformal energy to the vascular target, a traversing proton beam (see Fig. 1) was applied to a mouse arterial thrombosis model dosed with IONs to induce the CNR effect. This approach enabled a site-specific thrombolytic ION-nanoradiator effects at the target thrombus without delivering Bragg-peak energy to the vascular endothelium and normal tissue.

The chemical degradation and reduction of a thrombus by the ION-nanoradiator effect were achieved by CNR-mediated nanoscale energy deposition^19^, which was indicated by enhancement of reactive oxygen species (ROS) production or the emission of low-energy ICD electrons.

## Results

### Arterial occlusion via a thrombus and thrombus uptake of IONs

Thrombus formation and complete obstruction were indicated by the reduction or disappearance of the Doppler beat in the Doppler flow probe and confirmed by the flow void on MRI scans (details are provided in the Supporting Information [SI]). All animals exhibited complete occlusion within 10–20 min by attaching a 5% ferric chloride film to the carotid artery. Resection of the thrombosed artery revealed that the intravascular space was almost completely filled with thrombus, to which polymorphonuclear neutrophils were recruited, as reported previously^20^.

Perls’ staining in the ION uptake study revealed an inhomogeneous distribution in the thrombus at 24 hours post-injection, as shown in Fig. 2. Endothelium uptake of the FeCl_3_ ion in the FeCl_3_-treated vessel was absent according to Perls’ staining of AlCl_3_-treated vessels, demonstrating that the thrombus uptake of IONs did not occur in the vessel. The nanoparticles were not sufficiently taken up at either 30 minutes or 2 hours after injection. Thus, the time for proton irradiation was set at 24 hours after injection of the nanoparticles. The average amount of ION uptake in the thrombus tissue was estimated to be 2.3 mg/g tissue by subtracting the Fe concentration in the vascular surface-located FeCl_3_ from that in the whole resected vessel containing the thrombus.

### CNR-mediated thrombolytic treatment

Animals with arterial thrombi were classified into five different treatment groups: the PA-2 Gy (n = 5) and PA-4 Gy (n = 5) groups received protons alone in the absence of nanoparticles, with a plateau dose of either 2 Gy or 4 Gy, respectively; the CNR-2 Gy (n = 7) and CNR-4 Gy (n = 7) groups received 300 mg of Fe_3_O_4_/BW kg and a proton beam of either 2 Gy or 4 Gy, respectively; and one group (IONs alone, n = 5) received 300 mg of Fe_3_O_4_/BW kg without proton irradiation. In the case of the two CNR groups, an experiment was performed with two different batches of animals (five animals in each batch); considering loss of expired animals during model preparation or post-treatment, seven animals were included in each CNR group. Fe_3_O_4_ nanoparticles were injected intravenously 24 hours prior to proton irradiation.

Reperfusion was not observed after proton treatment alone at a dose of either 2 Gy or 4 Gy. Blood flow in the carotid artery was only restored in the CNR-4 Gy-treated mice (7 of 7), demonstrating that this treatment had complete efficacy in maintaining vascular patency, whereas reperfusion did not occur at all in the CNR-2 Gy-treated mice.

The average area of the arterial thrombus remaining after treatment is summarized in Fig. 3. The average value was 80% in the ION-only group, which is slightly less than baseline. More than 65% of the thrombus remained in a dose-dependent manner in the two proton-only groups, and less of the thrombus was observed in the PA-4 Gy-treated mice than in the PA-2 Gy group. The CNR-treated mice exhibited a significant reduction in the thrombus. The CNR-4 Gy-treated mice displayed a 60% reduction in the thrombus area, which was a significant (*p* < 0.01) reduction compared with that in the thrombi of the proton-only group (showing only a 20% reduction), as shown in Fig. 4A,B. In the CNR-2 Gy group, thrombus reduction was comparable (*p* < 0.05) to that of the CNR-4 Gy group, but was less than 50%. No vascular ruptures were found in the CNR-treated or proton-only mice. Distinctive structural alterations^21^ associated with radiation were not noted, even in the inner wall where the IONs were distributed very close or nearly attached to the wall in the CNR-4 Gy-treated mice, as shown in Fig. 4C.

### Vascular damage study of normal arteries

The safety of the vessel structures was also evaluated with relevant doses of traversing proton beam irradiation in separate proton treatments of normal mouse arteries. The inner elastic media and adventitia of the vessel were intact after exposure to a plateau proton dose of 2–5 Gy. Typical features of damage by X-ray irradiation^22^, such as neointimal proliferation, atheromatosis, thrombosis, and rupture, were not observed in the histologic analysis of normal carotid arteries that were irradiated with a plateau dose in the range of 1–10 Gy from a 100-MeV traversing proton beam (microscopy images are available in the SI).

### Enhanced production of ROS by proton-irradiated IONs

Water radiolysis by protons alone induces the generation of ˙OH, hydrogen radicals (H˙) and solvated electrons (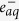) as the primary radiolysis products. In the presence of IONs, LEEs, including Auger electrons, Coulomb-ejected electrons, and ICD electrons, initiate Type I ROS generation by charge transfer to produce 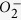 from dissolved oxygen molecules. Type II ROS generation is induced to a lesser extent by energy transfer from fluorescent X-rays to produce the same species, as in primary radiolysis.

Because 2-[6-(4-amino) phenoxy-3H-xanthan-3-on-9-yl] benzoic acid (APF) and hydroethidine-dihydroethidium (DHE) are highly specific to ˙OH and 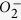, k respectively^23^, ION-enhanced ˙OH or 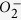 levels were measured using the increased slope of the fluorescence-irradiation dose in the presence of nanoparticles relative to that of the proton-alone irradiation curve, as demonstrated in Fig. 5A and B, respectively. These results are similar to the previously reported enhancement of gold nanoparticles treated with X-ray irradiation^23^. The ratios of the fluorescence enhancement of the slope in the phantom with 25-μM IONs to that for protons alone were 1.15 for ˙OH enhancement and 2.53 for 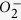 enhancement, suggesting 2-fold more LEE emission than X-ray fluorescence from the ION-nanoradiator.

### Electron emission yield by NEXAF analysis

NEXAF was measured by counting the emitted electrons with a kinetic energy of 3 eV to probe the potent enhanced electron density arising from the ICD-origin relaxation of Fe_3_O_4_ nanoparticles by photoexcitation with Fe L-edge X-rays. The electron yields from the photoelectrically ionized Fe_3_O_4_ nanoparticles were compared with those of a separate molecular species, FeCl_3_. Interestingly, a significant level of Shirley background was observed in the nanoparticle sample. For the 400-μg nanoparticle sample, the ratio of the Shirley background area to the area of the three peaks was 0.22, implying that for a single resonant electron, 0.22 additional electrons were generated as Shirley background; this value increased to 0.38 for the 600-μg sample. However, no notable Shirley background was observed in the spectra of the FeCl_3_ samples, as shown in Fig. 6.

### X-ray pump optical probe based on scanning transmission X-ray microscopy (STXM)

The fluorescence of the oxidant DHE caused by superoxide anions was localized to a single cell containing X-ray irradiated nanoparticles, as demonstrated by the red fluorescence shown in Fig. 7. In contrast, unexposed cells, which were a few tens of micrometres away from an exposed cell, did not fluoresce. Thus, limited transport of LEEs, only within a single cell dimension, occurs (10–20 μm). A small trace of red fluorescence appeared outside an exposed cell, which was likely secondary radiolysis of water by fluorescent X-rays rather than the transmembrane migration of the oxidant DHE.

## Discussion

The enhancement of thrombus reduction in the CNR-treated group suggests the presence of nanoradiator-mediated dose enhancement effects via the enhanced production of either electrons or ROS. Importantly, such dose enhancements were achieved by a traversing proton beam-induced ION-nanoradiator instead of Bragg-peak energy delivery. The high imprecision of conformal energy delivery limits its application to vascular diseases with thrombosis or atherosclerotic plaques because of potent mismatched delivery of a therapeutic Bragg-peak dose to the vascular endothelium and resultant vascular damage in conventional SOBP-based proton therapy. The main strategy in the present study was to induce site-specific dose enhancements only at the nanoparticle site of the thrombus without delivering a significant dose to the vascular structure by the traversing beam. In the traversing proton beam, a radiation dose of 10–20 Gy passed the carotid arterial body and neck tissue when a corresponding plateau dose of 2–4 Gy was deposited in the arterial tissue based on a 5:1 peak-to-plateau ratio at 100 MeV. The protons passing by the thrombus interacted with the ION, inducing a site-specific therapeutic nanoradiator effect^15^ and conserving the vascular endothelium via a low-LET of the plateau dose^24^. The resulting thrombolytic efficacy depended on the distribution and thrombus uptake of the nanoparticles. The restoration of the flow patency by the CNR treatment suggested the presence of thrombolytic activity along the entire affected vessel with thrombus uptake of nanoparticles despite the inhomogeneous volume distribution. The small number of IONs near the vessel wall and the restriction of the nanoradiator effect to approximately 10 μm from the nanoparticle site, as shown in Fig. 7, might result in a minimal effect on the vessel. A simulation study also revealed that proton-irradiated intraluminal gold nanoparticles exert relatively minimal effects on vessel walls^25^. Advanced damage, except for the partial infiltration of red blood cells, was absent in the proton-treated vessel, despite the endothelial pinocytic localization of FeCl_3_ ions. This may be explained by the relatively insignificant electron emission from FeCl_3_ compared to the thrombus uptake of Fe_3_O_4_ nanoparticles and by the minor endothelial denudation and collagen exposure resulting from FeCl_3_-treatment^26^.

Safety under a traversing proton beam is also related to the biodistribution of IONs, which was described in a review^27^ and in our previous report^15^. The blood half-life of IONs was one hour, which is relatively short, and the accumulation in healthy muscle was minimal (less than 5 μg Fe/g tissue) without significant side effects during a one-year follow up period^15^.

Measurement of ROS production provides insight into the composition of the CNR dose when considering the rapid conversion of LEEs and fluorescent X-rays to the superoxide anion or hydroxyl radical, respectively, in aqueous medium. Measurement of intracellular ROS, particularly in activated platelets, is important for understanding CNR-induced thrombolytic activity via oxidative stress in activated platelets. Fucoidan specifically binds with P-selectin, a molecular determinant of atherothrombotic disease^28^, in activated platelets. Fucoidan-conjugated IONs^29^ may improve homogeneous delivery of IONs to the thrombus, which facilitates the identification of the biological effects of redox-active IONs^30^,^31^ alone by targeting P-selectin, similar to protein disulphide isomerase in gold nanoparticle-mediated radiosensitization^32^,^33^. The efficacy of targeted delivery of IONs, together with the long-term efficacy and complications of CNR-based thrombolysis, will be studied further in clinical settings.

The approximately two-fold higher yield of superoxide anions relative to that of hydroxyl radicals suggests a relative yield ratio of electron emission to fluorescent X-rays by a similar factor as that from proton-irradiated Fe_3_O_4_ nanoparticles. Furthermore, a difference of 7.6 in the relative yield ratio of the electron emission to fluorescent X-rays from X-ray irradiated gold nanoparticles was noted^23^. The relative yields of electrons and X-ray photons may affect thrombolytic efficacy based on the chemical reactivity and transport of LEEs and fluorescent X-rays. For instance, longer wavelengths of fluorescent X-rays result from the Au-nanoradiator (13 keV or 68.4 keV) than from the Fe-nanoradiator (~7 keV), which may affect a relatively extended area. Dependence of the CNR dose on the Z-value and proton energy were studied separately and are under review for publication in another journal. Electronically excited atoms or molecules placed in a loosely bound chemical system (such as a hydrogen-bonded or van der Waals-bonded cluster) may efficiently decay by transferring their excess energy to neighbouring species that then emit LEEs^13^,^14^. This ICD process is a common phenomenon^34^,^35^,^36^,^37^ and a direct source of LEEs that may cause radiation damage either directly or via additional ROS production^38^,^39^,^40^,^41^,^42^. Because the atoms in mid/high-Z nanoparticle species are present in typical van der Waals-bonded cluster environments, electron emissions from proton-irradiated IONs occur via both Auger cascades at the directly ionized iron atoms and ICD paths at nearby neutral iron atoms^17^. The enhancement of the Shirley background in the electron yield from the ION films demonstrated that more LEEs could potentially be produced from the Fe-Fe ICD-present Fe_3_O_4_ nanoparticles via the ICD process than by Fe-Fe ICD-absent FeCl_3_. The ICD process in Fe_3_O_4_ nanoparticles emits enhanced background electrons with low kinetic energy, wherein the photons generated by the relaxation of core holes allow electrons delocalized within nanoparticles to escape from their bound state^43^. This process may explain why the FeCl_3_ sample, in which the binding energy of the localized outer shell electron was higher than that of the delocalized electrons in the iron nanoparticle, did not exhibit any meaningful background enhancement in its NEXAF spectra. However, variation in the Shirley background can readily be the subject of substrate artefacts including the surface condition of material film. Further studies are desirable to measure electron emission from nanoradiators using a nanoparticle streaming system devoid of substrate artefacts. The minimal therapeutic effects were obtained from *in vivo* studies with proton- or X-ray-irradiated cisplatin, in which the platinum concentration taken up in the tissue was 2.6 ± 17^44^ μg/g or 214 ± 17^45^ platinum μg/g, respectively. One may expect a better therapeutic effect from a potent platinum-nanoradiator-induced dose enhancement if the same amount of platinum was accumulated in the form of platinum nanoparticles.

Taken together, these observations suggest that CNR-mediated enhancement of the chemical degradation of a thrombus can be achieved directly via LEEs and indirectly via ROS formation in aqueous media. Therefore, chemical-based rheolytic mechanisms avoid the potentially serious complications that have been associated with distal embolization, haemolysis, and haemoglobinuria resulting from mechanical fragmentation or maceration of thrombi^4^.

## Methods

### Arterial thrombosis model

BALB/c mice (6–8 weeks old; 18–25 g; Samtako Bio Korea, Osan, Korea) were used in the present study. The animals were housed and cared for in accordance with the Guide for the Care and Use of Laboratory Animals. The procedures for the use of laboratory animals were approved by the Institutional Animal Care and Use Committee of Catholic University Hospital of Daegu (approval number, DCIAFCR-151007-7-Y). The arterial thrombosis model was prepared by attaching a 5% ferric chloride film to the exposed carotid artery of the mouse, as previously reported^20^ (details are described in the SI).

### Proton irradiation for CNR therapy

**Figure 1:**
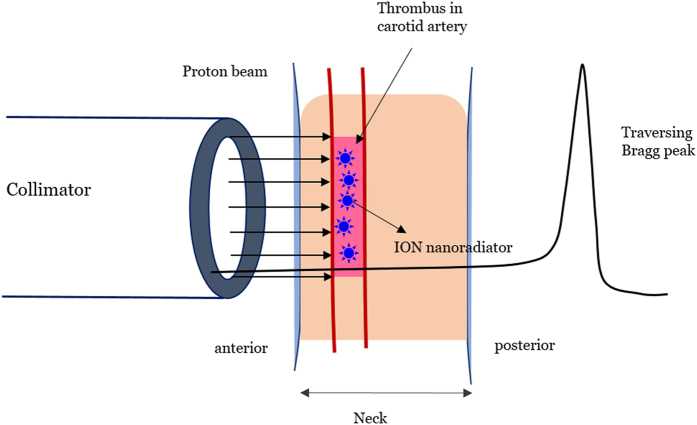
Schematic of the experimental procedure for the proton irradiation of a mouse arterial thrombus with a 100-MeV proton beam traversing the mouse neck. Bragg-peak energy was not deposited in the tissue penetrating depth, which was less than 25 mm. The beam was illuminated only on the thrombus tissue using a collimator with a diameter of 6 mm.

**Figure 2:**
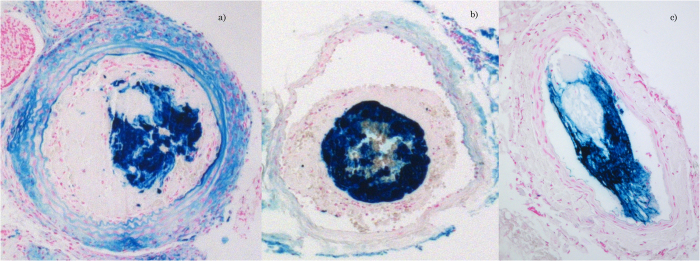
Perls’ Prussian blue staining of sectioned FeCl_3_-treated (**a,b**) or AlCl_3_-treated (**c**) arterial tissue when Fe_3_O_4_ nanoparticles were taken up in the thrombus 24 hours after the intravenous injection of 300 mg of Fe_3_O_4_/kg BW in an arterial thrombosis mouse model. IONs were injected 20 min after confirming thrombus formation with a Doppler flow probe.

**Figure 3:**
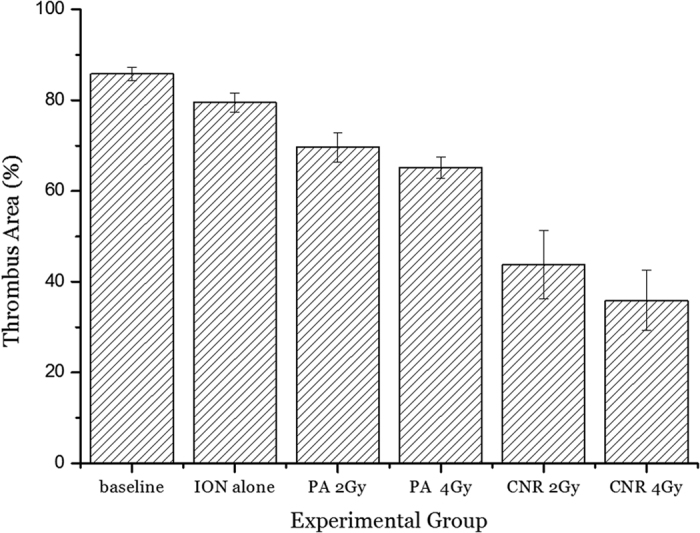
Average thrombus area measured at 7 days after treatment from the following treatment groups: baseline; untreated thrombosis mice as a basic control, IONs alone; thrombosis mice given only Fe_3_O_4_ nanoparticles, PA 2 Gy or 4 Gy; thrombosis mice given only proton irradiation with a plateau dose of 2 Gy or 4 Gy without nanoparticles, and CNR 2 Gy or 4 Gy; thrombosis mice given nanoparticles and proton irradiation with a plateau dose of 2 Gy or 4 Gy. The thrombolytic effect was statistically evaluated by comparing the thrombus area in each CNR-treated mouse group with the thrombus area for proton irradiation alone. The ION alone and two PA groups were additional control groups used to estimate the significance of CNR-mediated thrombolytic efficacy in the CNR groups. Differences among the groups were assessed by one-way analysis of variance (ANOVA) followed by Student’s *t*-test using a contemporary statistical software package (GraphPad Prism^TM^; GraphPad Software, Inc., San Diego, CA, USA). For all tests, a value of p < 0.05 was considered significant.

**Figure 4:**
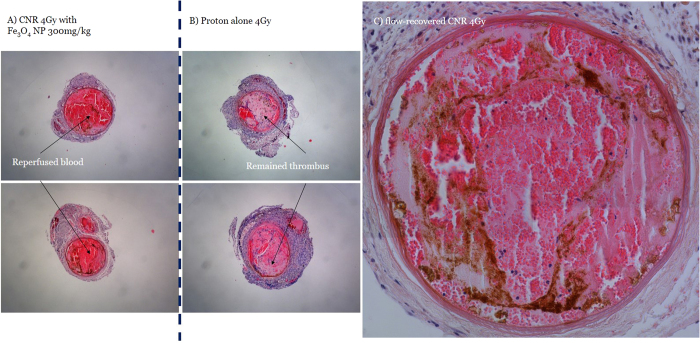
Haematoxylin/eosin (H/E)-stained sections showing a large portion (~72%) of the blood cells in the flow-recovered artery from a CNR 4 Gy-treated mouse (**a**) compared to the remaining occlusive thrombus in the artery from a proton-only 4 Gy-treated mouse (**b**). A typical flow-recovered artery from a CNR-4 Gy mouse that received 300 mg of Fe_3_O_4_/kg BW 24 hours prior to proton irradiation (**c**). The inhomogeneous distribution of IONs was demonstrated by the H/E staining of a sectioned artery. No structural damage, such as rupture or intimal thickening, was observed, despite the presence of nanoparticles near or attached to the wall with an inhomogeneous distribution.

**Figure 5:**
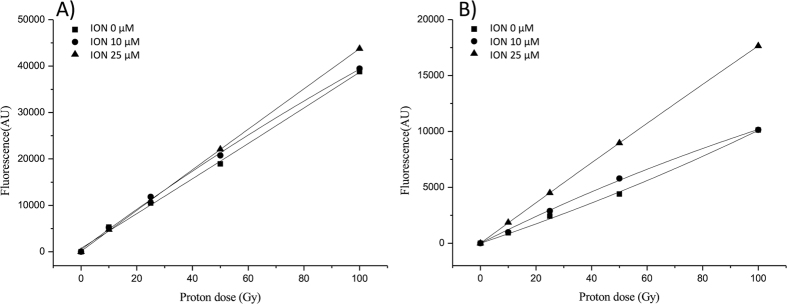
Fluorescence intensity of APF (**a**) and DHE (**b**) for 0-, 10-, and 25-μM Fe_3_O_4_ nanoparticle (ION) solutions in the presence of 100-MeV traversing proton irradiation. The relative enhancement ratios of the APF or DHE fluorescence levels between 0- and 25-μM Fe_3_O_4_ nanoparticle solutions at 50 Gy were estimated to be 1.15 and 2.53, indicating the relative yield enhancements of ˙OH or 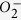, respectively.

**Figure 6:**
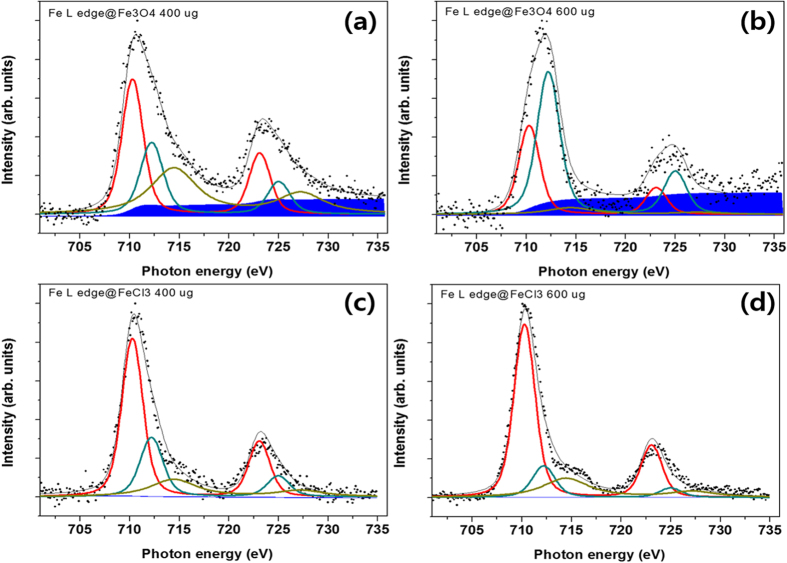
Fe L-edge NEXAF spectra of Fe_3_O_4_ nanoparticle films ((**a**) 400 μg; (**b**) 600 μg) and FeCl_3_ films ((**c**) 400 μg; (**d**) 600 μg) on a silicon-nitride window substrate. Electron yields with kinetic energies of 3 eV were measured relative to the Fe-Fe ICD-associated electron emissions from IONs, which revealed large differences in the Shirley background levels of the nanoparticles and separate molecular species. The points represent the raw data and the grey curve is a fit taking into account the different fitted oxidation state contributions, representing Fe^2+^ (710.3 eV, red curve), Fe^3+^ (712.5 eV, dense green curve) and Fe^2+^ satellite (714.1 eV, faint green).

**Figure 7:**
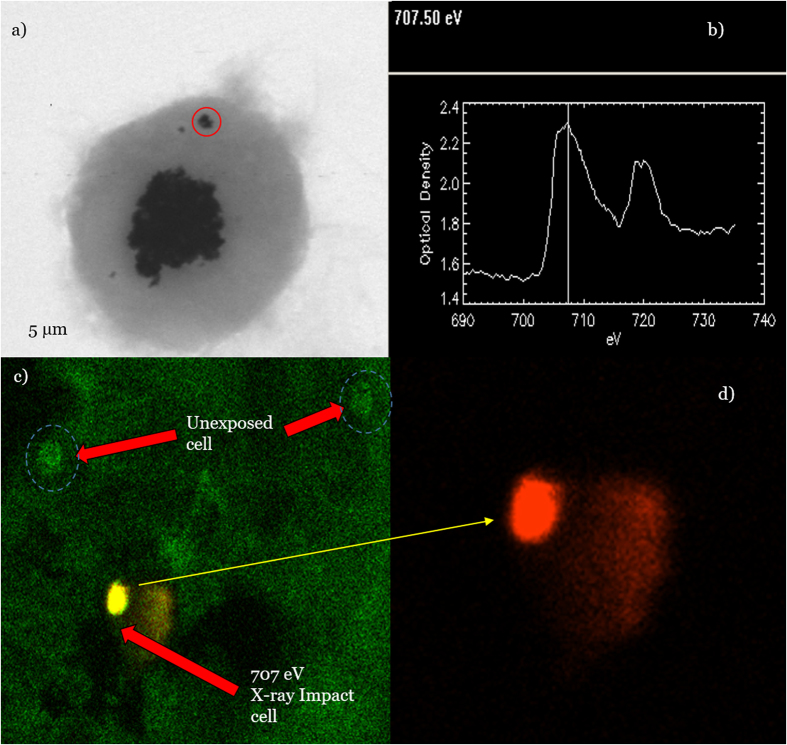
STXM image of a macrophage containing nanoparticles (red circle) selected for X-ray irradiation (**a**) and the corresponding 705 eV X-ray absorption spectrum (**b**) of the selected nanoparticle site (red circle). Oxidant-DHE fluorescence image of a macrophage containing IONs, which was irradiated using a 30 nm-focused X-ray photon of 707 eV. Photoelectric-absorption LEE emission demonstrated energy transfer to local oxygen, producing a distribution of 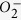 via unfiltered (**c**) and filtered red fluorescence (**d**) of the oxidant DHE in unexposed and exposed cells and in an exposed cell alone, respectively. The material was mostly confined within a single cell, but trace secondary radiolysis of water by fluorescent X-rays was feasible.

The animal model was subjected to 100-MeV proton beam irradiation using the experimental setup presented in Fig. 1 at the Korea Multipurpose Accelerator Centre (KOMAC, Kyungju, Korea). An average plateau proton dose of 2 Gy or 4 Gy was delivered to the neck tissue, including the thrombus, with a dose rate of 0.51–0.67 Gy/s. The proton beam that traversed through the mouse neck had a tissue-penetrating depth of less than 25 mm, producing a CNR effect without Bragg-peak deposition in the mouse neck.

The proton doses were measured at the entrance point of the mouse artery, and the Bragg-peak position (external mouse body) was determined using radiochromic film (MD-V3, Ashland, Covington, Kentucky, US) and read with a scanner (Epson Perfection V700 Photo). The mice were anesthetized with an intraperitoneal injection of 20 mg/kg ketamine and 18.4 mg/kg xylazine. Each group was irradiated with a single proton dose. The previously marked thrombus area was exposed to the proton beam via a collimator to avoid unwanted irradiation of the surrounding normal tissues. The flow patency was evaluated using a Doppler flow meter 7 days after treatment, and the mice were then euthanized. The treated vessels were removed and then subjected to fixation and staining for histologic analysis. The areas of the remaining thrombus from three different cross sectional planes (middle, proximal, distal; thickness of 20 μm) in the dissected vessels were measured using default image analysis software with an optical microscope (Axiophot, Zeiss, Germany), and the averaged value was recorded as the area value for each animal.

### ROS measurement of the nanoradiator effect

Water phantoms containing various concentrations of Fe_3_O_4_ nanoparticles (IONs, 0 μM, 10 μM, and 25 μM) and ROS probes, 5 μM APF or 100 μM DHE (Molecular Probes, Eugene, Oregon, USA) were irradiated by a 100-MeV traversing Bragg-peak proton beam with five different radiation doses (0 Gy, 10 Gy, 25 Gy, 50 Gy, and 100 Gy). For each nanoparticle concentration, three batches of samples in each phantom were treated with a given radiation dose. The fluorescence of the oxidants DHE or APF after reacting with superoxide or hydroxyl radicals, respectively, were measured immediately after removing the nanoparticles by centrifugation. The average values of the measured fluorescence in each phantom were plotted as a function of the irradiated proton dose. The slopes from the linear regression analysis were calculated, and the ratio of the fluorescence enhancement slope of the phantom with 25 μM IONs to that of the phantom with protons only was recorded as the relative yield of either superoxide or hydroxyl radicals.

### NEXAF study

Four samples of Fe_3_O_4_ nanoparticles and FeCl_3_ with two different densities (400 μg and 600 μg) were prepared by the precipitation method into the rectangular trench (1 × 1) of a silicon nitride X-ray window (Norcada, NX5050D, Alberta, Canada). To prepare a thin film of sample materials with maximum dispersion on the substrate, we evaporated the solvent of the aqueous sample solution slowly in a nitrogen environment oven at room temperature to minimize the aggregation effect. A well-dispersed part of the sample film was selected by optical microscopy prior to irradiation with the Fe L-edge X-ray. NEXAFS spectra of the Fe L-edge were obtained by counting the electrons with a kinetic energy of 3 eV. This electron voltage is in the energy range where bi-product electrons are generated owing to the ICD process. An electron analyser (R3000, Scienta) was utilized to detect electrons of 3 eV at each photon energy step of 0.1 eV. Data were recorded in a UHV chamber (2 × 10^−9^ Torr) at room temperature with an incident X-ray resolution of 0.1 eV. All spectroscopic measurements were conducted with the 4D photoemission beamline of the Pohang Accelerator Laboratory (PAL) in Korea. The curve fit to the spectra had three peaks for the oxidation states of Fe, representing the Fe^2+^ (710.3 eV), Fe^3+^ (712.5 eV), and Fe^2+^ satellite (714.1 eV), and the Shirley background.

### X-ray pump optical probe based on STXM

The macrophage uptake and intracellular distribution of IONs was imaged using synchrotron radiation based on the STXM technique in cells (1 × 10^4^) grown in 1 mg/mL IONs and 10 μM DHE in a silicon-nitride window (Norcada, NX5050D, Alberta, Canada) at the PAL 10 A bending magnet beamline (Pohang, Korea). Either cells or IONs were imaged briefly by performing raster scans across macrophage/iron nanoparticles at the peak O K-edge energy (542 eV) or Fe L-edge energy (707 eV), respectively. Selected IONs in a single cell were irradiated for 10 sec by X-rays (1.93 × 10^8^ photons) with an energy of 707 eV, and then the ROS-oxidant fluorescence of the DHE was measured using a confocal fluorescence microscope to detect the range of energies transferred to produce superoxide anions.

## Conclusion

The combination of a traversing proton beam and Fe_3_O_4_ nanoparticles produced a thrombolytic effect in an arterial thrombosis mouse model. This work demonstrated the flow-recovery ability of effectively reducing a thrombus via a site-specific therapeutic nanobeacon and provides opportunities for a new platform for particle therapy with extended therapeutic indications. This nanotechnology may have potential applications in the treatment of tumour infiltration, restenosis after vascular stent placement, cardiovascular vulnerable plaques, and other thrombotic occlusions when t-PA is ineffective.

## Additional Information

**How to cite this article**: Jeon, J.-K. *et al*. Coulomb nanoradiator-mediated, site-specific thrombolytic proton treatment with a traversing pristine Bragg peak. *Sci. Rep.* **6**, 37848; doi: 10.1038/srep37848 (2016).

**Publisher's note:** Springer Nature remains neutral with regard to jurisdictional claims in published maps and institutional affiliations.

## Supplementary Material

Supplementary Information
