# Supplementary material for: Coulomb nanoradiator-mediated, site-specific thrombolytic proton treatment with a traversing pristine Bragg peak
Source: Sci Rep. 2016 Nov 29;6:37848. doi: 10.1038/srep37848 (PMC5126678; doi:10.1038/srep37848)
Supplement: Supplementary Information [file srep37848-s1.doc]

**Coulomb nanoradiator-mediated, site-specific thrombolytic proton treatment with a traversing pristine Bragg peak**

Jae-Kun Jeon^a†^, Sung-Mi Han^b†^, Soon-Ki Min^c^, Seung-Jun Seo^a^, Kyuwook Ihm^d^, Won-Seok Jang^a^, and Jong-Ki Kim^a*^

***Synthesis of nanoparticles***

Alginate-coated Fe_3_O_4_ magnetite nanoparticles (IONs) were synthesized by insonating ferrous and ferric salt solutions, as reported previously (24). Briefly, FeCl_2_·4H_2_O (1.72 g) and FeCl_3_·6H_2_O (4.70 g) (8.65 mmol Fe^2+^/17.30 mmol Fe^3+^) were dissolved in 80 ml of distilled water. A black magnetic oxide precipitate was obtained by heating the solution to 80°C in an argon atmosphere, increasing the pH to 10 by adding 28–30% ammonium hydroxide and insonating the mixed iron solution with 20-kHz ultrasound at a power output of 140 W for 1 h. Alginate was used to coat the nanoparticle surfaces and disperse the particles. Briefly, 2 g of magnetite nanoparticles was dispersed in 60 ml of saline and 25 ml of 0.8% alginic acid solution by heating the solution to 80°C while insonating at a power output of 50 W for 30 min under nitrogen gas with continuous stirring.

The particles were purified by washing with saline with exposure to a strong neodymium magnet (magnetic field density; Br=11,000 Gauss). Finally, a ferrofluid containing 25-mg/ml IONs was obtained.


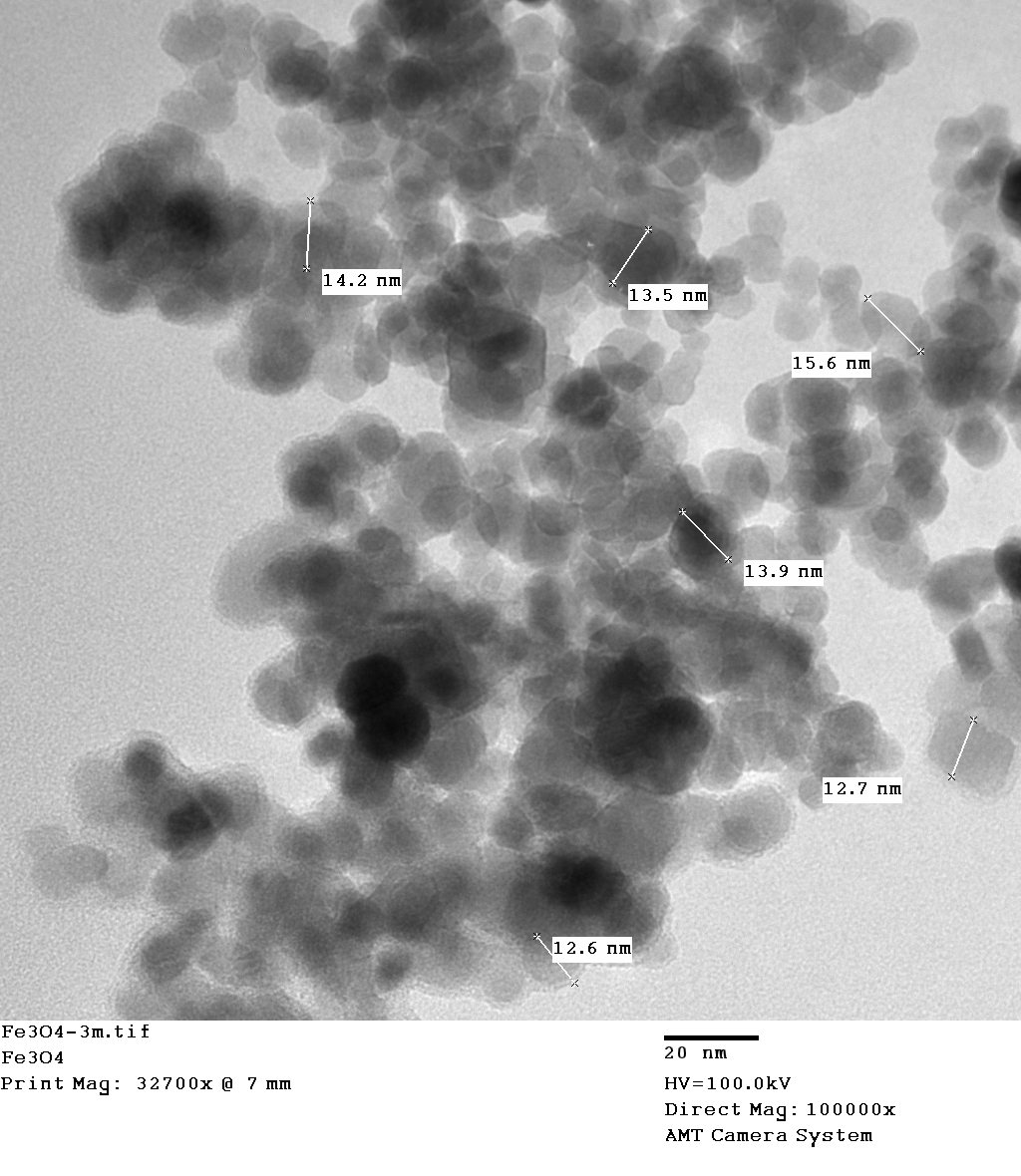


SI-Figure 1. The average particle size, size distribution and morphology of IONs were examined using a 902 transmission electron microscope (TEM; Carl Zeiss Pty Ltd, Oberkochem, Germany). The IONs were globular, with diameters of 10.6 ± 0.8 nm. After alginate coating, the IONs’ measured diameters were 13–15 nm.

***Arterial thrombosis model***

The mice were intraperitoneally anesthetized with 7.5 mg of ketamine hydrochloride (Hoffmann-La Roche, Basel, Switzerland) and 2.5 mg of xylazine per 100 g of body weight (BW). After anesthesia, an incision was made in the skin directly above the right common carotid artery region. The fascia was then bluntly dissected, and a segment of the left common carotid artery was exposed. Thrombosis was induced by applying two pieces of filter paper (12 mm) (Gel Blot Paper, GB003, Schleicher and Schuell, Keene, New Hampshire, USA) saturated with 5% FeCl_3_. The pieces of filter paper were placed on the opposite sides of the carotid artery (one beneath and one above), in contact with the adventitial surface of the vessel. The filter paper was applied for 3 min and then removed. Carotid blood flow was monitored with a miniature Doppler flow probe (Model 0.5 VB, Transonic System, Ithaca, New York, USA) after applying the filter paper. A cone-shaped ultrasonic gel was applied to the surface of the flow probe to maximize impedance matching while removing potent air contact between probe and artery. The time to occlusion, defined as the disappearance of the Doppler beat, was recorded. The excised skin was sutured after confirming occlusive thrombus, and the thrombus region was marked for further proton irradiation.

***Magnetic resonance (MR) imaging of the arterial thrombus model***

MR imaging was performed on selected mice using a 1.5-T MRI unit (GE 1.5 T, US) to image the flow void with the thrombus seven days after treatment with FeCl_3_. Axial T1-weighted images were acquired using a wrist coil and gradient echo sequence with the following scan parameters: a flip angle of 20, repetition time of 25 ms and echo time of 6.9 ms. Then, a fast spin echo (FSE) sequence was acquired to image the thrombus in the obstructed carotid artery 24 hours after intravenous injection of IONs at a dose of 300 mg/kg. The scan parameters of the T1-weighted FSE imaging were as follows: echo time of 15 ms, repetition time of 450 ms, field of view (FOV) of 80 mm, imaging matrix of 320×256, slice thickness of 2 mm, echo train length (ETL) of 4, and number of excitations (NEX) of 4.

**SI-Figure 2.** MRI of a carotid artery in an untreated normal mouse (a), the ferric chloride-induced thrombosis model in the right carotid artery (b), and corresponding maximum intensity projection (MIP) reconstructed image of the normal artery (d) compared with the flow void imaging (e). The T1-weighted FSE image after the injection of IONs revealed enhanced contrast in the occlusive thrombosis artery, probably because of the uptake of superparamagnetic nanoparticles by the thrombus (c).

***Thrombus uptake of nanoparticles***

At the time of arterial occlusion based on the disappearance of the Doppler beat, nanoparticles were intravenously injected with 300 mg of Fe_3_O_4_/kg BW via the tail vein. Thrombus uptake of nanoparticles was evaluated from the extracted vessels at designated times (30 min, 2 h, and 24 h post arterial occlusion) using Perls' Prussian blue staining. The blood vessel containing the thrombus was extracted by cutting both ends of the occluded part and fixing them. Histologic analysis of hematoxylin/eosin (H/E)-stained and Perls’ Prussian blue-stained sectioned tissues with a thickness of 10 μm was performed by optical microscopy. ION uptake was also evaluated using an inductively coupled plasma mass spectrometry (ICP-MS) instrument 24 hours after the intravenous injection of 300 mg of Fe_3_O_4_/kg BW.

***Safety test of the vascular endothelium under traversing proton beam irradiation***

Carotid arteries in normal mice (n=5) were irradiated with a 100-MeV traversing proton beam with the following plateau/Bragg peak doses: 2/10, 4/20, 6/30, and 10/50 Gy. Histopathologic examinations were performed by optical microscopy (Zeiss, Germany) of the exposed arteries after dissection and H/E staining one week after proton treatment.

Although this result was obtained from single temporal point, normal arteries exposed to the plateau dose of 1-10 Gy did not show neointimal proliferation, thrombosis and rupture. Plateau dose of traversing proton would have lower LET compared to Bragg-peak energy, resulting in minimal structural alteration in the vessel ^24^.

a)


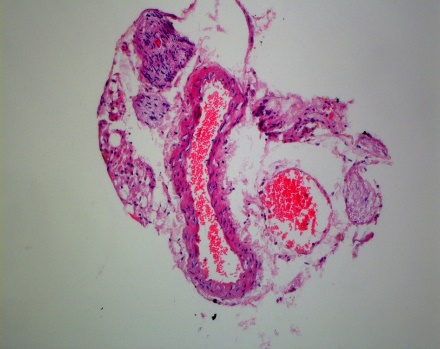

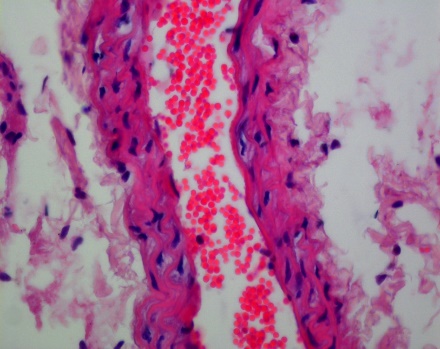

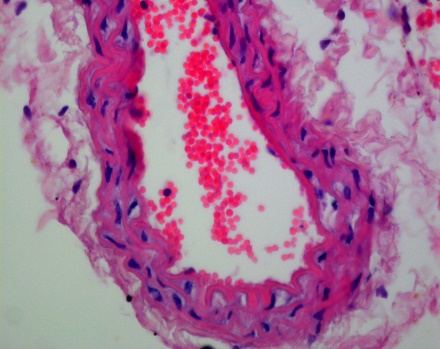


x10

x40

x40

b)


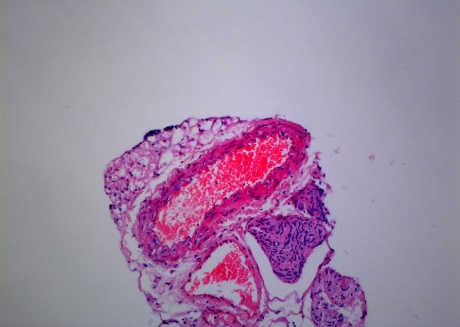

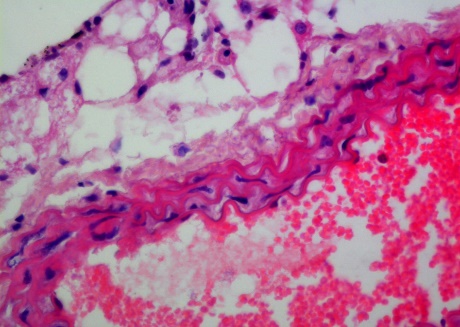

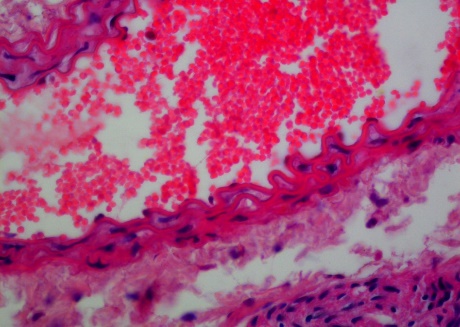


x10

x40

c)


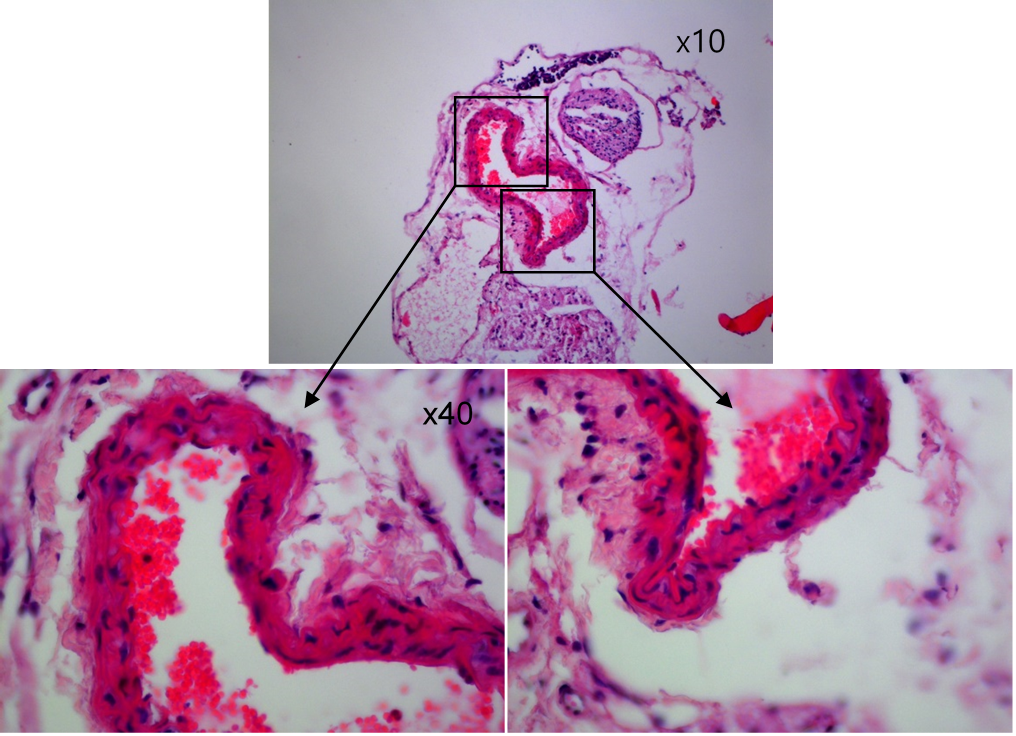


**d)**

**
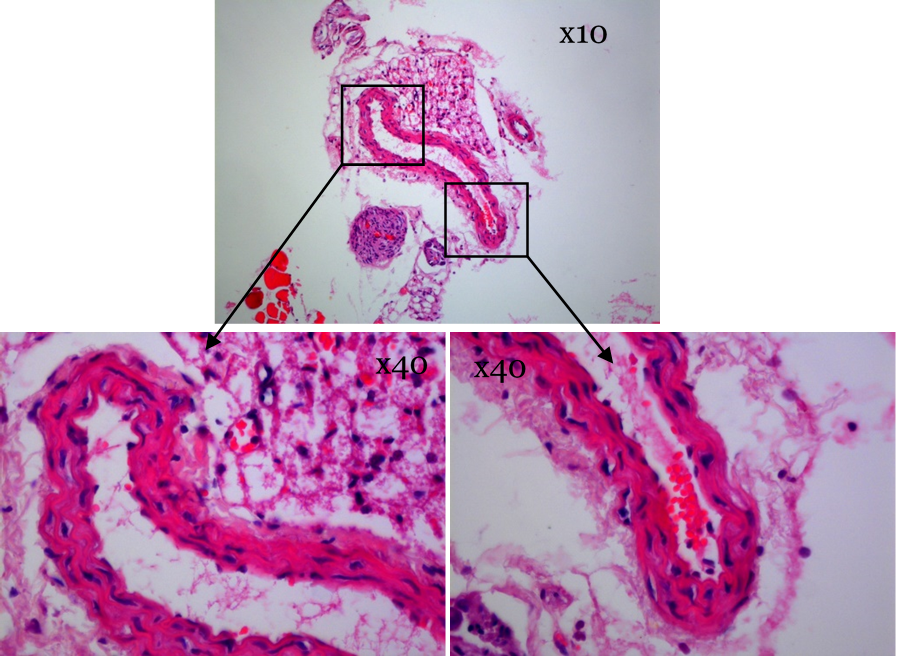
**

**SI-Figure 4.** A proton beam in the range of 1-10 Gy (plateau dose) that traversed a normal artery did not significantly damage the vascular structure. (a: 1 Gy, b: 2 Gy, c: 5 Gy, and d: 10 Gy)
